# Physicochemical and Structural Characterization of Composite Gels of Commercial Hemp Seed Protein Concentrate and Hemp Seed Protein Hydrolysate

**DOI:** 10.3390/gels12060484

**Published:** 2026-06-01

**Authors:** Dan Gao, Junqiang Huang, Zhenhua Duan, Qingli Xie, Yuthana Phimolsiripol, Pornchai Rachtanapun, Noppol Leksawasdi

**Affiliations:** 1 Guangxi Key Laboratory of Health Care Food Science and Technology, College of Food and Bioengineering, Hezhou University, Hezhou 542899, China; m18062542442@163.com (D.G.); 15077410524@163.com (J.H.); dzh65@126.com (Z.D.); 2 Center of Excellence in Agro Bio-Circular-Green Industry (Agro BCG) & Bioprocess Research Cluster (BRC), School of Agro-Industry, Faculty of Agro-Industry, Chiang Mai University, Chiang Mai 50100, Thailand; yuthana.p@cmu.ac.th (Y.P.); pornchai.r@cmu.ac.th (P.R.); 3 Faculty of Agro-Industry, Chiang Mai University, Chiang Mai 50100, Thailand; 4 Guangxi Key Laboratory of Optoelectronic Information Processing, School of Optoelectronic Engineering, Guilin University of Electronic Technology, Guilin 541004, China

**Keywords:** hemp seed protein concentrate, hemp seed protein hydrolysate, gel network, physicochemical and structural characterization

## Abstract

Hemp seed protein hydrolysate (HSPH), despite its high digestibility and solubility, exhibits severely impaired gelation properties due to extensive hydrolysis, thereby limiting its food applications. This study analyzed the effect of homogeneously incorporating commercial hemp seed protein concentrate (HSPC) into HSPH on physicochemical and structural properties of the resultant composite gels. As the HSPC concentration increased from 100 to 150 mg/mL, the composite gels exhibited a significant enhancement in hardness (*p* < 0.05), increasing from 1.63 to 5.74 N, along with an improvement in water-holding capacity (WHC) from 45.52 to 55.46%. Concurrently, the storage modulus (G′) and gelation temperature increased, with the latter rising from 65 to 78 °C. SDS-PAGE analysis suggested that the enhanced composite gel properties were attributed to its high-molecular-weight protein fractions (10–15 kDa and 40–50 kDa) of HSPC, which functioned as the primary structural components of the gel network. In addition, the formation of denser yet irregular microstructures was observed by scanning electron microscopy (SEM) analysis when HSPC incorporation increased from 0 to 200 mg/mL. Fourier-transform infrared (FTIR) further suggested that these improvements were due to increases in β-turn and random coil contents by approximately 9.60 and 7.73%, respectively. These findings provided insights into the utilization of HSPH and HSPC in plant-based foods and contributed to food security and sustainable agriculture.

## 1. Introduction

The global population is projected to attain 10.4 billion no later than 2100, posing unprecedented challenges to protein supply sustainability [1]. Concurrently, plant-derived proteins have garnered substantial interest due to economic, cultural, and environmental considerations. The worldwide plant protein market is anticipated to increase from USD 20.33 billion in 2025 to approximately USD 46.82 billion by 2035 [2]. This growth has intensified the valorization of agro-industrial by-products into sustainable protein alternatives [3].

The distinction between cannabis (a drug) and non-narcotic industrial hemp (*Cannabis sativa* L.) has captured interest in hemp seed products. Hemp seeds are a high-quality nutritional source, boasting hypoallergenic properties and highly digestible protein [4]. After oil extraction from hemp seeds, the resulting cake contains up to 50% protein, making it a valuable byproduct [5]. Hemp seed protein possesses all essential amino acids required by children (2–5 years old), including lysine, methionine, and tryptophan, as recommended by the FAO/WHO. Some research has focused on incorporating industrial hemp protein into various food formulations, such as gluten-free bread [6], gluten-free biscuits [7], meat products [8], and ice cream [9].

In food systems, gelation is one of the most critical techno-functional properties of plant proteins. Hemp seed protein concentrate (HSPC) and hemp seed protein hydrolysate (HSPH) constitute two primary commercial hemp protein products, with the former prepared via alkaline extraction–isoelectric precipitation and the latter produced through enzymatic hydrolysis. Under specific conditions, including thermal treatment, pH adjustment, and ionic strength modulation, hemp seed protein exhibits desirable gelling properties [5]. However, commercial HSPH typically exhibits inferior gelation properties due to its high degree of hydrolysis, as numerous studies have shown that excessive hydrolysis compromises the gel-forming capacity of plant proteins. This diminished gelling ability has been attributed to reduced surface hydrophobicity and shorter peptide chain lengths of hydrolysis products [10].

To date, extensive research has examined the gelation behavior of hydrolyzed plant proteins or modified gelling properties resulting from their complexation with polysaccharides and other components. For instance, soybean protein isolate hydrolysates (SPHs) were prepared through limited enzymatic hydrolysis with trypsin or papain, resulting in significantly increased solubility and gelation behavior [11]. Peanut protein isolate hydrolysates generated through limited Alcalase^®^ hydrolysis demonstrated a significantly enhanced gel-forming capacity at low temperature [12]. Quinoa protein isolate hydrolysates, obtained via enzymatic treatment with *Aspergillus niger* enzymes extracted from solid-state fermentation, exhibited substantially diminished mechanical strength in acid-induced gels [13]. An investigation on SPHs combined with locust bean gum revealed that a degree of hydrolysis (DH) exerted a pronounced influence on gel strength [14]. Furthermore, transglutaminase (TGase), a cross-linking agent, is effective in improving the gelation property of native and hydrolyzed soy protein [15]. Notably, low-DH soy protein hydrolysates exhibited superior gel behavior following TGase-mediated cross-linking, as evidenced by a homogeneous network architecture, enhanced viscoelasticity, and increased water-holding capacity [16].

Notably, research on the gelling properties of HSPH, whether alone or in composite formulations with other proteins, is scarce. Existing studies have primarily focused on whole hemp seed protein gels, investigating the effects of alkali-induced *Dendrobium officinale* polysaccharides [17], germination combined with ultrafiltration [18], probiotic fermentation [19], ionic strength, and pH [20].

To enhance the gelation ability of hemp protein peptides and broaden their application in food formulations, such as meat binders, fat replacers and plant-based meat analogs, HSPC was applied to improve the gel properties of HSPH. This homologous protein–peptide system preserves native protein content and intrinsic amino acid composition, ensures consistent digestibility, and mitigates allergenic risks associated with exogenous proteins. According to our preliminary experiments, HSPC could improve the gel ability of HSPH. Thus, this study investigated physicochemical and structural properties of the composite gels. The commercial HSPC and HSPH utilized in this study provided a more realistic representation of industrial-scale production conditions.

## 2. Results and Discussion

### 2.1. Proximate Composition Analysis and Techno-Functional Properties of HSPC and HSPH

#### 2.1.1. Proximate Composition Analysis of HSPC and HSPH

The proximate compositions of HSPC and HSPH are summarized in Table 1. HSPC exhibited a protein content of 66.63%, which was higher than that of commercial hemp seed protein concentrates (46.33% for organic and 47.72% for conventional) reported in the literature [21]. It also retained a considerable lipid fraction (12.64%), which was consistent with previous analyses [21] (10.87% for organic and 10.69% for conventional). However, HSPC exhibited lower carbohydrate content (8.76%) and higher ash content (11.97%) than those reported in the literature [21]. High standard deviations of carbohydrate content of HSPC and HSPH in Table 1 were due to the propagated analytical errors from protein, ash, and fat determinations. HSPC and commercial hemp protein concentrates differed in proximate composition, suggesting potential differences in raw material origin and processing conditions. The total protein content of HSPH was 62.08%. Compared with HSPC, HSPH demonstrated a 1.7-fold increase in the soluble protein fraction (52.47% vs. 30.78% in HSPC), confirming efficient peptide release during hydrolysis. Lipid and ash contents of HSPH exhibited lower values (1.45% and 4.62%, respectively), while carbohydrate content was substantially higher (31.85%). These compositional changes are critical because plant protein techno-functionality is fundamentally governed by compositional attributes, particularly the proportions of protein, carbohydrate, and lipid components [22]. These constituents modulate intermolecular interactions, which in turn regulate the gelation kinetics and network architecture of protein–peptide composite systems [23].

#### 2.1.2. The Techno-Functional Properties of HSPC and HSPH

The techno-functionality of plant proteins is intrinsically pH-dependent [24]. As presented in Table 2, the solubility of HSPH increased from 81.73% at pH 3.0 to a maximum of 94.49% at pH 7.0 and then decreased to 85.15% at pH 9.0. The decreased solubility at pH 9 might result from alkali-induced conformational unfolding, exposing hydrophobic regions that promote peptide aggregation by hydrophobic interactions or disulfide bonding [25]. Although most of the hydrolyzed peptides maintain high solubility across a broader alkaline range, the solubility of microalgal hydrolysates (*Chlorella* sp. and *Scenedesmus* sp.) was relatively limited, with values ranging from 57.33% to 65.65% across the tested pH range (2–10) [26]. It could be suggested that the solubility of the hydrolyzed peptides was influenced by the protein sources. In contrast, the solubility of HSPC increased progressively with pH, particularly in the alkaline range, rising from 38.62% at pH 7.0 to a maximum of 83.87% at pH 11. These results were consistent with a reported study [27], which showed that hemp seed protein isolate (HSPI) exhibited improved solubility across the pH range of 4 to 9.

Emulsifying and foaming properties are two key functional characteristics directly governed by the interfacial activity of proteins [28]. In this study, both HSPC and HSPH exhibited negligible foaming capacity. The emulsifying activity index (EAI) of HSPC and HSPH was markedly low at different pH levels, ranging from 0.0025 to 0.0051 m^2^/g. In a previous study, HSPI also exhibited limited emulsifying capacity, with markedly low EAI values [18]. The emulsifying stability index (ESI) was measured 10 min after emulsion formation. Given the low initial EAI values, the resulting ESI data were not statistically meaningful. Despite HSPH’s substantially higher solubility compared to HSPC, its emulsifying properties were not significantly improved. The diminished molecular size of hydrolyzed peptides has been identified as a limiting factor in the formation of cohesive, viscoelastic boundary films at oil droplet interfaces [29,30].

The water-holding capacity (WHC) of both HSPC and HSPH powders remained relatively stable across most pH conditions tested. The WHC values of HSPC were comparable to or lower than those of HSPI and commercial hemp seed concentrates in previous studies [18,21]. Notably, HSPH powder exhibited significantly higher WHC values than HSPC powder (*p* < 0.05) (Table 2). Such improvements probably arise from greater exposure of hydrophilic residues following protein denaturation, together with higher carbohydrate content in HSPH, both promoting water retention [22].

Reliable gel-forming capability in plant proteins is essential for developing viable alternatives to animal-derived gelled foods. HSPC at 20% (*w*/*v*) formed stable gels at pH 5.0 and 7.0, which was lower than the reported gel-forming concentration of 22% [27]. In contrast, HSPH at any tested pH and HSPC at pH 3, 9 and 11 failed to form gels, which exhibited a fluid-like behavior. Consistently, HSPC samples demonstrated substantially higher WHC than HSPH samples, particularly at pH 5.0 and 7.0. In this study, the pH of HSPC suspensions in deionized water was 7.79. The suspension exhibited spontaneous gelation without pH adjustment, confirming robust gelation under native conditions. Therefore, all HSPC-HSPH composite gels prepared in the following study were produced without pH adjustment to enhance industrial applicability.

### 2.2. Characterization of Gels

#### 2.2.1. TPA

**Figure 1:**
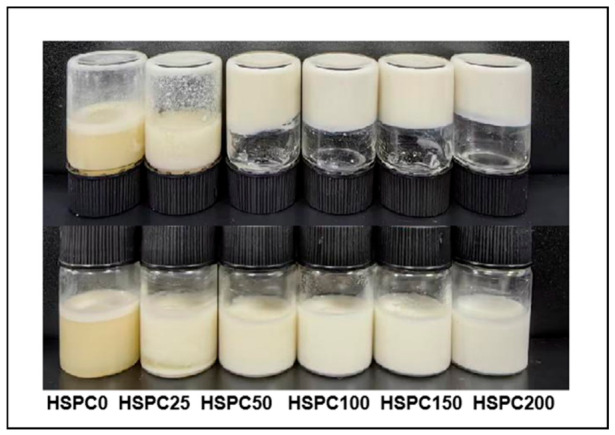
Images of HSPC0, HSPC25, HSPC50, HSPC100, HSPC150 and HSPC200 gels.

**Figure 2:**
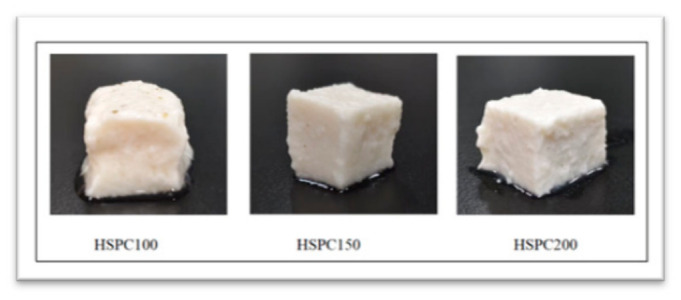
The visual appearance of tofu-like gels of HSP100, HSP150, and HSP200.

In this study, the gel samples were designated as HSPC0 (pure HSPH), HSPC25, HSPC50, HSPC100, HSPC150, and HSPC200 (pure HSPC), corresponding to HSPC concentrations of 0, 25, 50, 100, 150, and 200 mg/mL, respectively. Their images are shown in Figure 1. Overall, the gel structures of HSPC0 and HSPC25 were not self-supporting and exhibited poor stability, requiring containment to maintain their structure. HSPC100, HSPC150, and HSPC200 demonstrated good structural stability, as shown in Figure 1 and Figure 2.

Hardness, often quantified as the peak stress a sample can withstand during compression, fundamentally represents a sample’s resistance to localized plastic deformation [31]. In this study, HSPC0 and HSPC25 exhibited fluid-like behavior, indicating insufficient network formation. Gel formation occurred when the HSPC concentration increased to 50 mg/mL. As demonstrated in Table 3, the hardness values of HSPC50, HSPC100, HSPC150, and HSPC200 were 1.17, 1.63, 5.74 and 17.05 N, respectively, exhibiting a progressive increase with HSPC concentration. This enhancement was mechanistically ascribed to the high-molecular-weight proteins in HSPC. These high-molecular-weight proteins were critical for establishing the robust network structure required for both gelation and superior water retention. SEM images of gel samples further confirmed the formation of denser networked architecture as the HSPC concentration increased (Section 2.2.5). Figure 2 shows the visual appearance of tofu-like gels of HSP100, HSP150, and HSP200. Although the pure HSPC gel exhibited a compact and stable network structure, its surface morphology was characterized by a coarse texture with more visible particulate aggregates. In contrast, the incorporation of HSPH resulted in a gel matrix with a smoother surface and finer internal structure (Figure 2, HSPC150). These findings demonstrate that the HSPC-HSPH combination not only compensates for the weak gelation of pure HSPH but also refines the coarse texture of pure HSPC gel, yielding a composite gel with improved surface smoothness and microstructural uniformity.

Springiness serves as an indicator of gel elasticity [32]. Cohesiveness quantifies the extent to which a gel maintains its structural integrity upon secondary deformation relative to its initial resistance. Chewiness denotes the mechanical work required to comminute a solid matrix to a swallowable state, which increases concomitantly with increased springiness and hardness. In this study, increasing HSPC concentration resulted in a significant increase in springiness, cohesiveness (except at the highest concentration) and chewiness (*p* < 0.05). These improvements in mechanical properties are practically relevant. Gel products with better cohesiveness can withstand greater mechanical stress, thereby retaining their structural integrity throughout the production chain.

#### 2.2.2. Chromatic Properties and WHC of the Gels

The product color directly influences consumers’ willingness to purchase. The chromatic properties of the composite gels were assessed by L* (lightness), a* (red–green), b* (yellow–blue), and W* (whiteness index) values, as presented in Table 4. The gel formed by HSPH exhibited a lightness of 64.21, a slight green hue of −2.81, and moderate yellowness of 5.58, corresponding to a visually yellowish appearance. With increasing HSPC addition, the L* of HSPC100 and HSPC150 significantly increased to 71.45 and 73.32, respectively. The b* of HSPC25, HSPC50, HSPC100, and HSPC150 also increased by 51.3, 90.1, 103.0, and 111.8%, respectively. Regarding the a*, HSPC25 displayed a slight green hue (−2.54), while HSPC50, HSPC100, HSPC150, and HSPC200 shifted to a red hue (0.073–0.82). The whiteness of the gels significantly increased (*p* < 0.05) when HSPC addition rose from 50 to 200 mg/mL, which was likely attributable to the intrinsic whiteness of HSPC. This dominated the optical properties of the composite gels. In addition, the whiteness and WHC of gels showed a similar trend. This might be attributed to the lightening effect of increased water content on the gel surface [33,34]. As HSPC concentration increased, the gel color became progressively whiter, which could be more advantageous for certain food applications.

The WHC of gels is a critical functional property in gelled products [35]. As shown in Table 4, HSPC0 (pure HSPH) exhibited a WHC of 11.49%. The WHC of the composite gels increased compared to HSPC0, possibly due to the existence of high-molecular proteins in HSPC. This indicates the enhanced capability of forming dense structures that could entrap water and was consistent with the increasing hardness values as shown in Table 3.

#### 2.2.3. Low-Field Nuclear Magnetic Resonance (LF-NMR) Analysis

**Figure 3:**
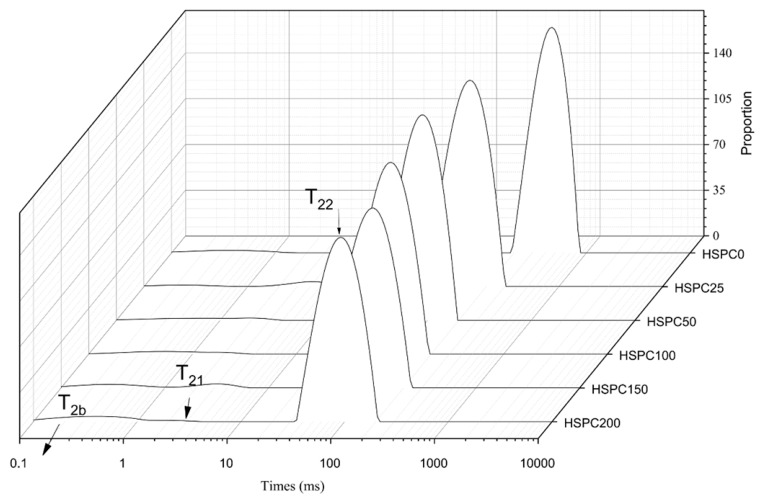
T_2_ relaxation time distributions of HSPC0, HSPC25, HSPC50, HSPC100, HSPC150 and HSPC200 gels.

LF-NMR is a technique to evaluate water distribution and mobility in food systems. LF-NMR can analyze different types of water in gels and their binding capacity by measuring relaxation times. Figure 3 shows the T_2_ relaxation-time distributions of the samples. T_2b_ (0.1–1 ms) corresponded to strongly bound water tightly associated with macromolecular components. T_21_ (1–10 ms) corresponded to weakly bound or immobilized water loosely associated with macromolecules. T_22_ (70–300 ms) corresponded to free water, physically entrapped by the protein gel network and was able to flow freely within the confined regions.

As presented in Table 5, HSPC0 exhibited a combined bound-immobilized water fraction (T_2b_ + T_21_) of 6.77% with the free water fraction (T_22_) comprising 93.23% of the total water content. Upon incorporation with HSPC, free water fractions of the samples all exceed above 95%. This phenomenon was analogous to the mechanical entrapment dominance observed in black bean protein gel systems where network density rather than molecular binding served as the primary water-retention mechanism [36]. At higher HSPC concentrations (150–200 mg/mL), the proportion of bound-immobilized water fraction (T_2b_ + T_21_) increased while T_22_ decreased, indicating stronger protein–water interactions and a reduction in free water content. This transformation indicated that HSPC facilitated the conversion of free water to a more tightly bound state by reinforcing gel integrity and gel density. The enhanced structural framework can restrict water mobility more effectively, thereby improving WHC through a combination of mechanical entrapment and increased binding sites.

#### 2.2.4. Rheological Analysis

Rheological temperature-sweep experiments were conducted to analyze the changes in storage modulus (*G*′) and loss modulus (*G*″) of the samples to evaluate their viscoelastic behavior during gelation. During thermal scanning, proteins gradually unfolded, leading to exposure of hydrophobic groups and subsequent gel formation at elevated temperatures [15].

**Figure 4:**
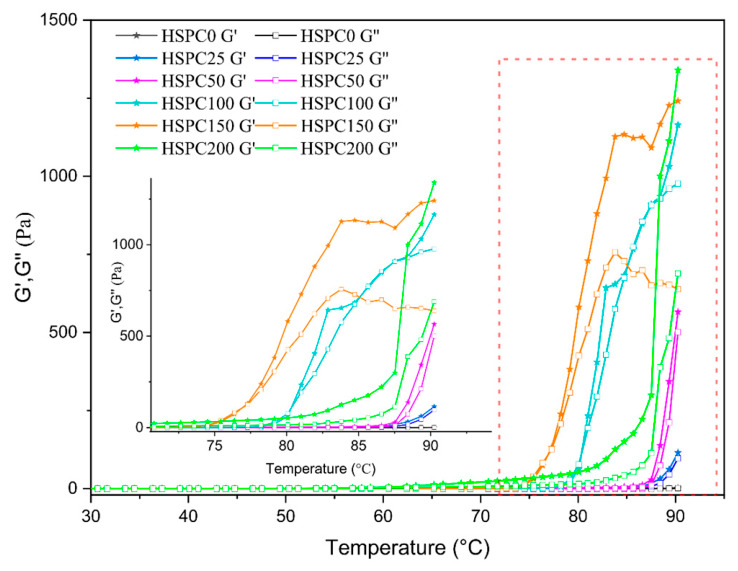
Rheological analysis of HSPC0, HSPC25, HSPC50, HSPC100, HSPC150 and HSPC200 gels. *G*′ and *G*″ represent storage modulus and loss modulus, respectively. Inset picture is a zoomed view of the area indicated by the red dashed box.

Both *G*′ and *G*″ of HSPC0 were close to zero and exhibited negligible changes during heating (Figure 4). When the HSPC concentration increased to 25 and 50 mg/mL, *G*′ and *G*″ increased slightly but remained at relatively low values. These samples behaved as free-flowing viscous liquids with absence of a dense gel structure formation. As HSPC concentration increased to 50, 100, and 150 mg/mL, *G*′ and *G*″ increased significantly, which suggested the formation of a stronger gel network.

Furthermore, the *G*′ values at 90 °C for different samples could be ranked in the ascending order as follows: HSPC0 < HSPC25 < HSPC50 < HSPC100 < HSPC150 < HSPC200, demonstrating that the addition of HSPC effectively enhanced the gel properties of the samples. The gelation temperature was determined from the crossover point of *G*′ and *G*″. As the HSPC concentration increased, the gelation temperature rose concomitantly. The gelation temperatures of HSPC100, HSPC150, and HSPC200 were approximately 65, 78, and 80 °C, respectively. Beyond the gelation point, both *G*′ and *G*″ of the gels elevated simultaneously. However, once *G*′ surpassed *G*″, the gel network structure began to form, indicating that the sample transitioned toward a solid-like state. At the gelation point, proteins or peptide molecules commenced unfolding, and intermolecular interactions between protein and peptide increased. The formation of aggregates and the sol–gel transition were subsequently observed [37].

#### 2.2.5. Microstructural Characterization

**Figure 5:**
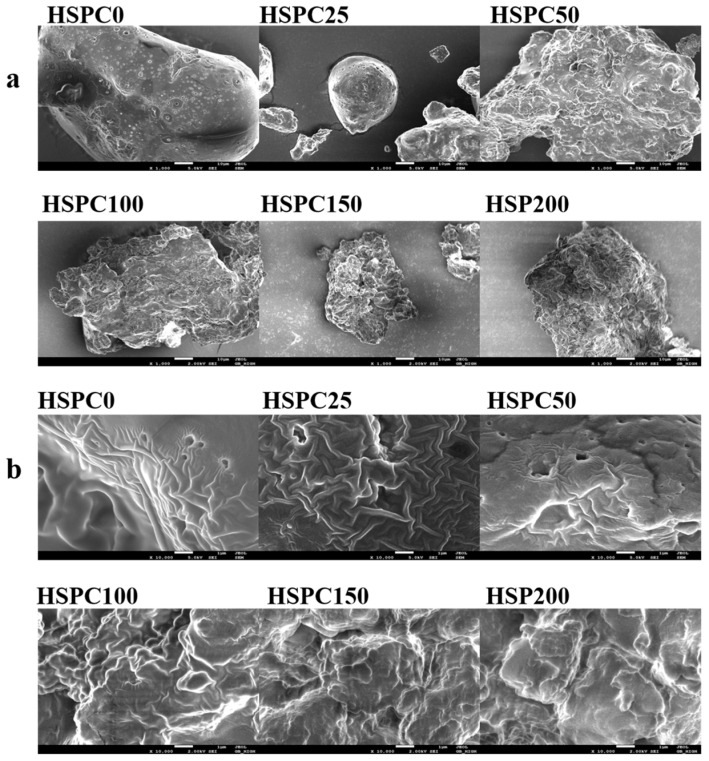
Microstructural characterization of gels by SEM at 1000-fold magnification (**a**) and 10,000-fold magnification (**b**).

Microstructural features can provide an insight into the development of gel network formation and structural organization. At ×1000 magnification (Figure 5a), HSPC0 (pure HSPH) and HSPC25 exhibited spherical particles with relatively uniform and smooth surfaces. The HSPC50–HSPC200 showed rough and uneven surfaces with wrinkles and indentations. At ×10,000 magnification (Figure 5b), HSPC0 and HSPC25 displayed large pores. The gel matrix of HSPC50–HSPC200 appeared increasingly dense, exhibiting smaller pores and a more compact architecture. Although a previous study demonstrated that gels with uniform pore distributions and homogeneous structures exhibited better WHC [38], this study revealed that the gels with progressively disordered structure exhibited better WHC and increased G′, as shown in Table 4 and Figure 5. As shown in Table 6, the disordered structures of the gels are likely attributed to the elevated β-turn and random coil contents with increasing HSPC concentration.

### 2.3. Structural Properties

#### 2.3.1. Sodium Dodecyl Sulfate-Polyacrylamide Gel Electrophoresis (SDS-PAGE)

**Figure 6:**
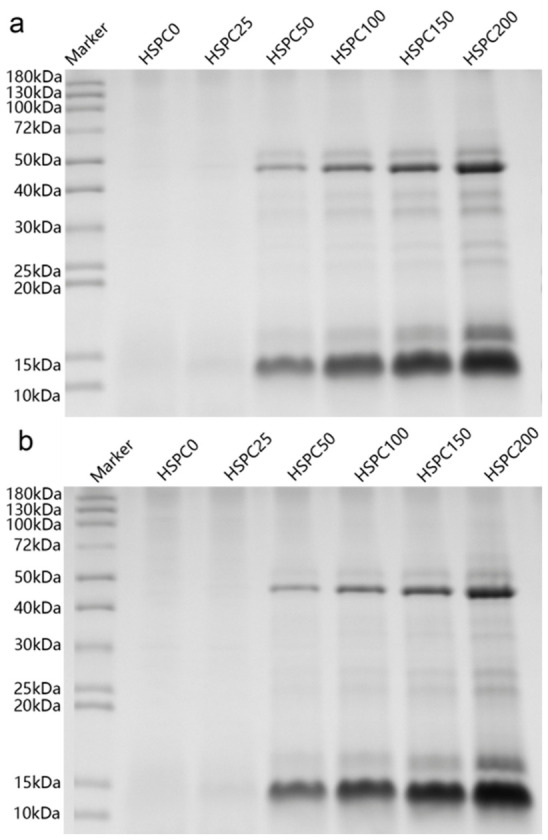
SDS-PAGE profiles of HSPC0, HSPC25, HSPC50, HSPC100, HSPC150 and HSPC200 gels ((**a**): non-reducing electrophoresis; (**b**): reducing electrophoresis).

The SDS-PAGE profiles of HSPH and HSPC are shown in Figure 6, in which Figure 6a and Figure 6b represent non-reducing and reducing conditions, respectively. The limited visibility of HSPH bands on SDS-PAGE was attributed to the extensive hydrolysis of hemp protein into small peptide fragments (<10 kDa), which typically exhibited poor retention within SDS-PAGE gels. Regarding HSPC, protein bands in the range of 10–15 kDa exhibited the highest abundance, followed by bands at 40–50 kDa. This was consistent with a study showing that the hemp milk preparation yielded prominent bands at approximately 48–54 kDa and 10–15 kDa under non-reducing conditions. These bands were assigned to two predominant storage proteins, namely globulin and albumin [39], respectively. Specifically, as reported [40], the protein bands at approximately 40–50 kDa, 30–40 kDa, and 20–25 kDa may correspond to vicilin (47 kDa), edestin acidic subunit (33 kDa), and edestin basic subunit (18–22 kDa). The increased gelation capacity of samples from HSPC25 to HSPC200 was attributed to the proteins about 10–15 kDa and 48–54 kDa. These high-molecular-weight proteins were essential for forming a dense gel network, primarily through hydrogen bonding, hydrophobic interactions, and covalent cross-linking.

#### 2.3.2. Surface Hydrophobicity (H_0_) Analysis

**Figure 7:**
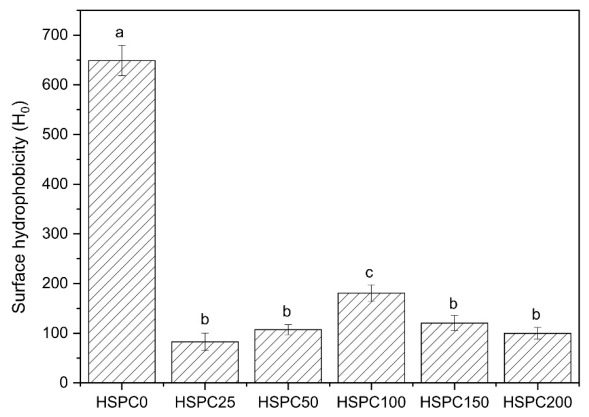
The surface hydrophobicity (H_0_) of HSPC0, HSPC25, HSPC50, HSPC100, HSPC150 and HSPC200 gels. Values with different letters differ significantly (*p* < 0.05).

*H*_0_ represents the relative surface exposure of hydrophobic amino acid residues. It is associated with protein solubility, emulsification capacity, and gel-forming property [12]. As presented in Figure 7, the *H*_0_ of the control hemp protein hydrolysate was 648.82. Upon the addition of HSPC, the *H*_0_ of the samples (HSPC25, HSPC50, HSPC100, HSPC150, and HSPC200) decreased by 87.26, 83.48, 72.14, 81.45, and 84.62%, respectively. The decrease indicated that hydrophobic interactions promoted molecular association, thereby burying hydrophobic residues within the gel network interior. This observation was consistent with findings in soy-protein-based gels, in which the decrease in *H*_0_ was attributed to the development of protein aggregates [41].

#### 2.3.3. Fluorescence Intensity

**Figure 8:**
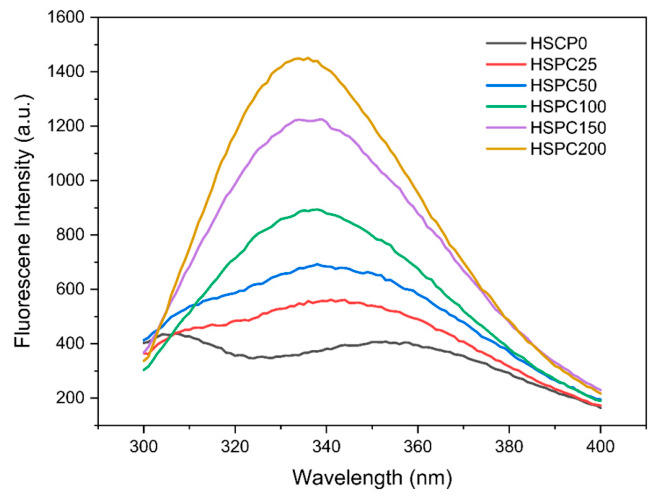
The intrinsic fluorescence spectra of HSPC0, HSPC25, HSPC50, HSPC100, HSPC150 and HSPC200 gels.

As tryptophan and tyrosine residues are sensitive to the microenvironment, changes in intrinsic fluorescence are widely used to monitor tertiary structural variations [13]. As shown in Figure 8, the HSPC0 showed two maximum emission wavelengths (*λ*_max_) at 303 and 350 nm, corresponding to tyrosine and tryptophan residues [42], respectively. With the addition of HSPC from 25 to 200 mg/mL, the λ_max_ of tryptophan exhibited a blue shift to 344, 338, 335, 334, and 336 nm, suggesting enhanced hydrophobicity of the microenvironment surrounding tryptophan residues. In addition, the fluorescence intensity of HSPC was 407.7. After the addition of HSPC, the fluorescence intensities of HSPC25, HSPC50, HSPC100, HSPC150, and HSPC200 increased by 37.70, 69.86, 118.4, 200.2, and 355.9%, respectively. The enhanced fluorescence intensity was possibly due to the inherently higher fluorescence quantum yield of HSPC and variations in tryptophan content, rather than increased surface exposure of hydrophobic residues [43].

#### 2.3.4. Secondary Structure and Functional Groups

**Figure 9:**
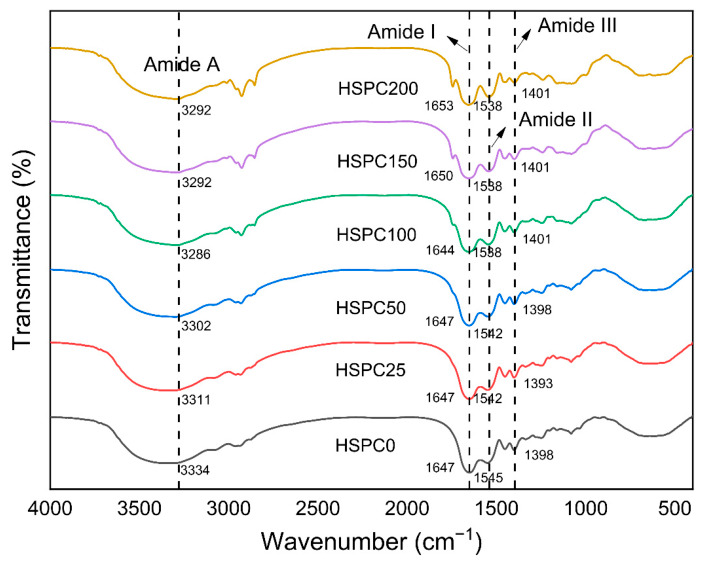
The FTIR spectra of HSPC0, HSPC25, HSPC50, HSPC100, HSPC150 and HSPC200 gels.

FTIR is a frequently used technique to determine the secondary structure of proteins and peptides. The FTIR spectra of HSPC and HSPH in Figure 9 exhibited characteristic protein-associated vibrational bands at 3334 and 3292 cm^−1^ (amide A, N-H stretching), 1647 and 1653 cm^−1^ (amide I, C=O stretching), 1545 and 1538 cm^−1^ (amide II, N-H bending), and 1398 and 1401 cm^−1^ (amide III), respectively. A progressive shift in amide A bands toward lower wavenumbers, accompanied by band narrowing, was observed as HSPC concentration increased (Figure 9), demonstrating strengthened hydrogen bonding interactions [17]. These spectral changes confirmed that structural reorganization occurred during gelation, leading to strengthened intermolecular interactions, network cohesion, as well as gel-forming capacity.

Analysis on the secondary structure of the gel samples was carried out by deconvolving the amide I absorption band (1600–1700 cm^−1^) recorded via FTIR spectroscopy (Table 6). The main secondary structure of HSPC was random coils subsequent to β-turns, while the major constituent of HSPH was α-helices and β-sheets. A similar observation was also reported in [17], where the β-turns dominated the secondary structure, followed by β-sheets in the HSPC gel. In this study, as the HSPC concentration increased, the α-helix and β-sheet contents showed decreased value. The β-turn and random coil contents showed increased value. This was consistent with a previous study suggesting that higher levels of disordered conformations can enhance protein solubility and further facilitate the formation of more well-organized gel network structures [44,45].

## 3. Conclusions

As compared with pure HSPH, the HSPC-HSPH composite gels exhibited markedly improved hardness and WHC. The enhanced gelation was attributed to high-molecular-weight protein fractions serving as structural backbones, coupled with strengthened hydrophobic interactions and increased disordered secondary structures (β-turns and random coils) as revealed by FTIR and fluorescence analyses. Although the composite gels showed lower mechanical strength than the HSPC200 (pure HSPC) gel, the composite gel of HSPC150 possessed a finer texture, smoother surface, and more uniform microstructure, which may facilitate its potential industrial applications such as meat analogs or dairy alternatives. These products may serve as viable vegan substitutes, offering a valuable option for individuals following plant-based or animal-free diets.

## 4. Materials and Methods

### 4.1. Materials

HSPC and HSPH were provided by Shaanxi Fengqiwu Biotechnology Co., Ltd. (Xi’an, China). β-mercaptoethanol and 1-anilinonaphthalene-8-sulfonic acid (ANS) were purchased from Aladdin Chemical Reagent Co. (Shanghai, China). The soybean oil (Jinlongyu, first-grade press) was bought from supermarket. Other chemicals were of analytical grade. The chemicals and the BCA™ Protein Assay Kit were obtained from Nanjing Jiancheng Bioengineering Co., Ltd. (Nanjing, China).

### 4.2. Nutritional Composition and Functional Properties of HSPC and HSPH

#### 4.2.1. Determination of Nutritional Composition

The proximate analysis of HSPC and HSPH were analyzed and expressed on a dry basis. The protein content was analyzed by the Kjeldahl method (GB 5009.5-2025) [46]. The crude fat content was analyzed by the Soxhlet extraction method (GB 5009.6-2016) [47]. The ash content was analyzed by the residue on ignition method by a muffle furnace (FO411C, Yamato Scientific, Tokyo, Japan) heated to 550 °C for 16 h (GB 5009.4-2016) [48]. The starch content was analyzed by the national standard method GB 5009.9-2023 [49]. Carbohydrate content was estimated by the difference method:

Carbohydrate content (g/100 g) = 100 − (protein + ash + crude fat)

#### 4.2.2. Protein Solubility

A previous method was used to determine the solubility of HSPC and HSPH [50]. Briefly, 1.0 mg/mL protein solutions were prepared and magnetically stirred for 1 h. Then, the pH of the solutions was adjusted to desired values (pH 3.0, 5.0, 7.0, 9.0, and 11.0) using either 1.0 M HCl or 1.0 M NaOH and stirred for 1 h. The pH values were rechecked and readjusted if necessary to ensure the desired pH level. The solutions were centrifuged at 4000× *g* for 15 min using a centrifuge machine (L535-1, Xiangyi Laboratory Instrument Development Co., Ltd., Changsha, China). The soluble protein in supernatant was determined by a BCA™ Protein Assay Kit. The determination of protein solubility was based on calculating the percentage of soluble protein relative to the total protein.

#### 4.2.3. Water-Holding Capacity (WHC) of HSPC and HSPH

The HSPC and HSPH powders with different pH values were prepared as follows. The protein solutions were set to pH 3.0, 5.0, 7.0, 9.0 and 11.0, using either 1.0 M HCl or 1.0 M NaOH. The solutions were stirred for 1 h at room temperature and then freeze-dried (−80 °C, 48 h) to powders. Dried HSPC and HSPH solutions (10%, *w*/*w*) were prepared to analyze their WHC [51]. The samples were subjected to centrifugation at 4500× *g* (20 min) after 30 min stirring. Following removal of the supernatant fraction, the residual precipitate was subjected to weighing measurement. WHC was defined as grams of water held by each gram of protein sample.

#### 4.2.4. Foaming Capacity and Foaming Stability

Foaming capacity (FC) and foaming stability (FS) of HSPC and HSPH were determined [52]. Sample solutions (0.2 g/mL) were prepared and adjusted to pH 3.0, 5.0, 7.0, 9.0, and 11.0 by 1.0 M HCl or 1.0 M NaOH. They were stirred overnight at 25 °C using a magnetic stirrer to ensure complete hydration. Then, 20 mL of each solution was prepared in a 50 mL tube. They were homogenized (10,000 rpm, 1 min) by a high-speed homogenizer (FJ-200, Shanghai Specimen Model Co., Shanghai, China). FC was calculated as the percentage volume increase:

FC (%) = (*V*_1_ − *V*_0_)/*V*_0_ × 100

where *V*_0_ and *V*_1_ refer to the sample volumes measured prior to and following the homogenization process, respectively.

After standing at 25 °C for 30 min, the foam volume was recorded. FS value was determined based on the proportion of foam volume that remained stable over this period.

FS (%) = *V*_2_/*V*_1_ × 100

where *V*_2_ represents the total volume after 0.5 h.

#### 4.2.5. Emulsifying Properties of HSPC and HSPH

Sample solutions of HSPC and HSPH (10 mg/mL) were adjusted at different pH values (3.0, 5.0, 7.0, 9.0, and 11.0) by 1.0 M HCl or 1.0 M NaOH and magnetically stirred overnight at 25 °C to ensure complete hydration. Emulsions were obtained by homogenizing (10,000 rpm, 2 min) 2 mL soybean oil and 6 mL sample solution. EAI and ESI were evaluated according to the reported method [52]. Then, 200 μL of emulsion from the bottom of the tube was diluted with 5 mL 1 mg/mL SDS solution. The absorbance of the dilute emulsion was determined at 500 nm by an UV–Vis spectrophotometer (VIS-722, Shanghai Youke Instrument Co., Ltd., Shanghai, China). The absorbance was determined again after 10 min. EAI and ESI were determined as follows:

EAI (m^2^/g) = (2 × 2.303 × *A*_0_ × *DF*)/(*c* × *θ* × 10,000)

ESI (min) = *A*_0_/(*A*_0_ − *A*_10_) × 10

where *A*_0_ and *A*_10_ are defined as the absorbances measured at the time points of 0 and 10 min. *DF* represents the dilution factor (26), *c* refers to the protein concentration (0.01 g/mL), and *θ* is the volume fraction of oil (0.25).

#### 4.2.6. Preparation of Gels

For the gels of HSPC and HSPH at different pH levels, the sample suspensions (200 mg/mL) were prepared at a series of pH levels (pH 3.0, 5.0, 7.0, 9.0, and 11.0) and stirred for 1 h at 25 °C. The mixtures were heated at 90 °C for 60 min, then cooled and stored in a refrigerator (4 °C) overnight.

For the composite gels, an HSPH suspension (200 mg/mL) prepared under the same conditions served as the control. Mixed systems were formulated with HSPC concentrations ranging from 25 to 200 mg/mL while maintaining a total protein concentration of 200 mg/mL. The mixtures were stirred for 60 min (25 °C) and incubated at 90 °C for 60 min, then cooled and stored in a refrigerator (4 °C) overnight. Samples were designated as HSPC0 (pure HSPH), HSPC25, HSPC50, HSPC100, HSPC150, and HSPC200 (pure HSPC) corresponding to HSPC concentrations of 0–200 mg/mL.

### 4.3. Characterization of Gels

#### 4.3.1. Colorimetric Evaluation of Protein Gels

The color parameters of gels were evaluated by a colorimeter (CR-400, Konica Minolta, Tokyo, Japan) operating in the CIE L*a*b* color system with a D65 light illuminant [53]. Three color parameters of L* (lightness), a* (red–green), and b* (yellow–blue) were obtained from the system. Calculation of the whiteness index (W*) for all protein gel samples was performed using the formula listed as follows [33]:

$$W^{*}\;=\;100\;-\sqrt{\left[{\left(100\;-\;L^{*}\right)}^{2}\;+{\;a}^{*2}\;+{\;b}^{*2}\right]}$$

#### 4.3.2. Texture Profile Analysis (TPA)

The textural properties of the gel samples were analyzed by a texture analyzer (TA.XT Plus, Stable Micro Systems Ltd., Godalming, Surrey, UK) equipped with a P/36R probe [54]. TPA mode was applied. The pre-test speed, test speed, and post-test speed were set to 5, 1, and 5 mm/s, respectively. The compression ratio, holding time, and trigger force were 75%, 5 s, 0.049 N, respectively. All measurements were conducted at 25 °C.

#### 4.3.3. WHC of Gels

The WHC was analyzed by a previously reported method with minor modifications [19]. The gel sample (5 g) was centrifuged at 4000× *g* (10 min). The supernatant was discarded, and the residual gel in the tube was determined. WHC was determined as follows:

$$\mathrm{WHC}(\%)\;=\frac{W_{3}-W_{1}}{W_{2}-W_{1}}\;\times \;100$$

where *W*_1_ refers to the weight of the empty centrifuge tube (g), *W*_2_ refers to the total weight of the tube together with the gel sample before centrifugation (g), and *W*_3_ refers to the weight of the tube containing the gel after the supernatant was removed (g).

#### 4.3.4. Low-Field Nuclear Magnetic Resonance (LF-NMR)

The water distributions of the gels were assessed by an NMR instrument (MacroMR12-150H-I, Suzhou Niumag Analytical Instruments Co., Ltd., Suzhou, China). The gel sample was prepared in a glass tube (11.6 mm diameter × 32 mm height). According to a previously reported method [55], the Carr–Purcell–Meiboom–Gill (CPMG) pulse sequence was applied as follows: waiting time (TW) = 1000 ms, number of scans (NSs) = 8, and number of echoes (NECHs) = 8000. The number of iterations was fixed at 10^4^.

#### 4.3.5. Rheological Testing During Gelation

The *G*′ and G″ of the gel samples were measured by a rheometer (Discovery HR-1, TA Instruments, New Castle, DE, USA) according to a previously reported method with slight modifications [56]. The sample suspension was placed between the parallel plates (40 mm diameter) with a 0.6 mm gap. The temperature was initiated from 25 to 90 °C at 10 °C/min. During the temperature sweep, the frequency was fixed at 1 Hz.

#### 4.3.6. Microstructure Analysis

The surface morphology of the samples was examined by SEM (scanning electron microscopy) using a previously reported method [57]. The surface morphology of the gel samples was observed with an SEM (JSM-7610F, JEOL Ltd., Tokyo, Japan) at magnifications of 1000× and 10,000×. Freeze-dried samples were carefully placed on double-sided adhesive tape, mounted on an aluminum specimen holder. A thin gold layer was subsequently deposited onto the surface to suppress charging effects under the electron beam. The sputtering conditions were set as a sputtering current of 30 mA and a sputtering time of 90 s. The photographed conditions were set as an accelerating voltage of 5 kV, a working distance of 15 mm and a chamber pressure of 9.5 × 10^−5^ Pa.

### 4.4. Structural Properties

#### 4.4.1. SDS-PAGE

The protein profiles were analyzed by SDS-PAGE under both non-reducing and reducing conditions following a previously reported method [58]. Briefly, the samples (2 mg/mL) were mixed with SDS-PAGE loading buffer. For the reducing conditions, β-mercaptoethanol (5%, *v*/*v*) was added. The reducing samples were subjected to heat treatment (95 °C, 10 min) before loading. The electrophoresis gel consisted of a 5% stacking gel and a 12% separating gel. Protein samples (7 μL) and commercial molecular weight marker (5 μL, 10–180 kDa) were injected into gel wells. The electrophoretic separation was performed at 150 V for about 90 min. Then, the gels were stained with a solution containing 0.3% (*w*/*v*) Coomassie Brilliant Blue R-250, 20% (*v*/*v*) isopropanol, and 10% (*v*/*v*) glacial acetic acid. Later, the gels were destained in a solution composed of 10% (*v*/*v*) isopropanol and 10% (*v*/*v*) glacial acetic acid.

#### 4.4.2. Surface Hydrophobicity (H_0_) Analysis

The *H*_0_ of the gel samples was determined according to a previous method [59]. First, gel samples were dispersed in 10 mM phosphate (Na_2_HPO_4_/NaH_2_PO_4_) buffer (2 mg/mL, pH 7.0) and homogenized by vortexing for 2 min. After being centrifuged (4000× *g*) for 15 min, the supernatant was used as the stock solution. The protein content of the supernatant was measured as 4.2.2. Next, five concentrations of each sample (0.02, 0.05, 0.10, 0.25, and 0.50 mg/mL) were obtained by diluting the supernatant with the same buffer. The 8 mM stock solution of ANS was dissolved in the same phosphate buffer (10 mM, pH 7.0). The mixed solution containing 20 μL of 8 mM ANS and 4 mL of protein solution was incubated for 30 min under light-protected condition. Subsequently, the fluorescence intensity was measured at 390 nm excitation and 470 nm emission with 5 nm slits by a fluorescence spectrometer (F-7000, Hitachi High-Tech Corp., Tokyo, Japan). *H*_0_ was determined based on the slope obtained from the linear regression analysis of fluorescence intensity versus protein concentration.

#### 4.4.3. Fluorescence Intensity

The intrinsic fluorescence spectra were recorded by a fluorescence spectrophotometer (F-7000, Hitachi Corp., Tokyo, Japan) [60]. First, gel samples were dispersed in 10 mM phosphate buffer (1 mg/mL, pH 7.0). Then, the intrinsic fluorescence of the sample solution was measured at 280 nm excitation, with emission recorded from 300 to 400 nm. The scanning speed of the fluorescence spectrum was 2400 nm/min. The slit widths for excitation and emission were set at 5 nm and 2.5 nm, respectively.

#### 4.4.4. FTIR Analysis

FTIR spectra of the gel samples were recorded over the wavenumber range of 400–4000 cm^−1^ range by an ATR-FTIR spectrometer (Nicolet iS5, Thermo Fisher Scientific, Waltham, MA, USA) [3]. Each spectrum was an average of 16 scans at a resolution of 4 cm^−1^. The wavenumbers of amide I, amide II, and amide III regions were determined by the peak-picking function of the OMNIC software (version 9.7) after baseline correction and smoothing.

### 4.5. Statistical Analysis

All experiments were performed in triplicate. All experimental values are shown as mean ± standard deviation (SD). Statistical significance was evaluated by one-way ANOVA with Tukey’s multiple comparison test (*p* < 0.05) using SPSS 26.0 (IBM Corp., Armonk, NY, USA). All figures were generated by OriginPro 2018 (64-bit) software (OriginLab Corp., Northampton, MA, USA).

## Figures and Tables

**Table 1:** Proximate composition analysis of HSPC and HSPH.

Proximate Composition (%)	HSPC	HSPH	Hemp Protein Concentrates [21]	
			Organic	Conventional
Total Protein	66.63 ± 0.50 ^b^	62.08 ± 1.06 ^a^	46.33 ± 0.40	47.72 ± 0.15
Total Lipid	12.64 ± 1.82 ^b^	1.45 ± 0.29 ^a^	10.87 ± 0.12	10.69 ± 0.13
Ash	11.97 ± 0.86 ^b^	4.62 ± 1.23 ^a^	8.76 ± 0.08	8.21 ± 0.22
Total Carbohydrates	8.76 ± 2.85 ^a^	31.85 ± 1.76 ^b^	34.05 ± 0.23	33.38 ± 0.46
Starch	0.13 ± 0.001 ^a^	0.29 ± 0.002 ^b^	0.82 ± 0.03	1.11 ± 0.03
Soluble Protein	30.78 ± 1.34 ^a^	52.47 ± 2.71 ^b^	-	-

Note: values with different letters between HSPC and HSPH differ significantly (*p* < 0.05).

**Table 2:** The techno-functional properties of HSPC and HSPH.

Samples	Solubility (%)	EAI (m^2^/g)	ESI (min)	WHC of Powder (g/g)	Gel Ability	WHC of the Gel (%)
HSPH						
pH 3.0	81.73 ± 0.92 ^d^	0.0039 ± 0.0006 ^c^	19.94 ± 3.53 ^bc^	0.97 ± 0.009 ^c^	−	9.71 ± 0.59 ^a^
pH 5.0	84.25 ± 0.10 ^e^	0.0051 ± 0.0001 ^d^	35.87 ± 3.97 ^f^	0.99 ± 0.02 ^c^	−	11.13 ± 0.65 ^b^
pH 7.0	94.49 ± 1.33 ^g^	0.0037 ± 0.0007 ^bc^	25.78 ± 2.61 ^de^	0.98 ± 0.01 ^c^	−	16.72 ± 0.45 ^c^
pH 9.0	85.15 ± 0.48 ^e^	0.0025 ± 0.0003 ^a^	30.46 ± 4.33 ^e^	0.97 ± 0.01 ^c^	−	16.69 ± 0.88 ^c^
pH 11.0	89.49 ± 1.22 ^f^	0.0036 ± 0.001 ^bc^	24.75 ± 2.03 ^cd^	0.95 ± 0.04 ^c^	−	19.92 ± 0.41 ^d^
HSPC						
pH 3.0	33.82 ± 1.39 ^a^	0.0051 ± 0.0004 ^d^	29.17 ± 2.73 ^de^	0.84 ± 0.007 ^b^	−	48.25 ± 0.75 ^f^
pH 5.0	36.67 ± 0.29 ^b^	0.0038 ± 0.0002 ^bc^	24.25 ± 2.75 ^bcd^	0.84 ± 0.02 ^b^	+	56.96 ± 0.47 ^h^
pH 7.0	38.62 ± 0.45 ^b^	0.0029 ± 0.0001 ^ab^	14.05 ± 1.25 ^a^	0.86 ± 0.01 ^b^	+	52.13 ± 1.00 ^g^
pH 9.0	57.03 ± 2.23 ^c^	0.0038 ± 0.0002 ^bc^	14.11 ± 0.25 ^a^	0.84 ± 0.02 ^b^	−	48.72 ± 0.46 ^f^
pH 11.0	83.87 ± 0.92 ^e^	0.0047 ± 0.0001 ^d^	19.28 ± 0.77 ^b^	0.77 ± 0.02 ^a^	−	42.64 ± 0.88 ^e^

Note: ‘+’ indicates gel formation; ‘−‘ indicates no gel formation. EAI, ESI, and WHC represent emulsifying activity index, emulsifying stability index, and water-holding capacity, respectively. Values with different letters in the same column differ significantly (*p* < 0.05). Only HSPC can form gels at pH 5 and pH 7. For other samples, ‘WHC of the gel’ reflects WHC of the fluid-like samples.

**Table 3:** Texture profile analysis (TPA) of HSPC50, HSPC100, HSPC150 and HSPC200 gels.

Samples	Hardness (N)	Springiness	Cohesiveness	Chewiness (N)
HSPC50	1.17 ± 0.059 ^a^	0.12 ± 0.01 ^a^	0.46 ± 0.02 ^a^	0.06 ± 0.002 ^a^
HSPC100	1.63 ± 0.101 ^b^	0.20 ± 0.03 ^a^	0.66 ± 0.03 ^b^	0.21 ± 0.028 ^a^
HSPC150	5.74 ± 0.247 ^c^	0.34 ± 0.01 ^b^	0.64 ± 0.02 ^b^	1.24 ± 0.098 ^b^
HSPC200	17.05 ± 0.404 ^d^	0.68 ± 0.12 ^c^	0.44 ± 0.03 ^a^	5.11 ± 1.045 ^c^

Note: TPA parameters could not be measured for HSPC0 (pure HSPH) and HSPC25 due to their fluid-like behavior. Values with different letters in the same column differ significantly (*p* < 0.05).

**Table 4:** Chromatic properties and WHC of HSPC0, HSPC25, HSPC50, HSPC100, HSPC150 and HSPC200 gels.

Samples	L*	a*	b*	W*	WHC (%)
HSPC0	64.21 ± 1.84 ^a^	−2.81 ± 0.19 ^a^	5.58 ± 1.33 ^a^	63.64 ± 1.59 ^a^	11.49 ± 0.29 ^a^
HSPC25	64.44 ± 0.37 ^a^	−2.54 ± 0.43 ^a^	8.44 ± 0.90 ^b^	63.36 ± 0.57 ^a^	21.29 ± 0.50 ^b^
HSPC50	64.88 ± 1.02 ^a^	0.073 ± 0.12 ^b^	10.61 ± 0.64 ^c^	63.30 ± 0.79 ^a^	30.28 ± 0.74 ^c^
HSPC100	71.45 ± 0.27 ^b^	0.56 ± 0.038 ^c^	11.33 ± 0.28 ^c^	69.28 ± 0.15 ^b^	45.52 ± 0.36 ^d^
HSPC150	73.32 ± 0.30 ^bc^	0.84 ± 0.091 ^c^	11.82 ± 0.075 ^c^	70.80 ± 0.25 ^b^	55.46 ± 0.30 ^e^
HSPC200	75.29 ± 0.76 ^c^	0.82 ± 0.045 ^c^	10.90 ± 0.11 ^c^	73.07 ± 0.66 ^c^	66.42 ± 0.45 ^f^

Note: values with different letters in the same column differ significantly (*p* < 0.05). The L*, a*, b*, W*, and WHC represent lightness, red–green, yellow–blue, whiteness index and water-holding capacity of gels, respectively.

**Table 5:** T_2_ relaxation times in HSPC0, HSPC25, HSPC50, HSPC100, HSPC150 and HSPC200 gels.

Samples	T_2b_ (%)	T_21_ (%)	T_2b_ + T_21_ (%)	T_22_ (%)
HSPC0	1.99 ± 0.42 ^bc^	4.77 ± 0.11 ^f^	6.77 ± 0.39 ^c^	93.23 ± 0.39 ^a^
HSPC25	0.91 ± 0.05 ^a^	2.10 ± 0.09 ^e^	3.02 ± 0.07 ^b^	96.99 ± 0.07 ^c^
HSPC50	1.76 ± 0.32 ^ac^	1.04 ± 0.19 ^b^	2.79 ± 0.25 ^a^	97.21 ± 0.25 ^c^
HSPC100	2.37 ± 0.39 ^c^	0.51 ± 0.08 ^a^	2.88 ± 0.33 ^a^	97.12 ± 0.33 ^c^
HSPC150	2.38 ± 0.14 ^c^	1.78 ± 0.19 ^d^	4.10 ± 0.11 ^b^	95.84 ± 0.11 ^b^
HSPC200	4.35 ± 0.70 ^d^	0.46 ± 0.16 ^a^	4.51 ± 0.56 ^b^	95.20 ± 0.56 ^b^

Note: values with different letters in the same column differ significantly (*p* < 0.05). T_2b_, T_21,_ and T_22_ represent strongly bound water, weakly bound or immobilized water, and free water, respectively.

**Table 6:** Secondary structure composition of HSPC0, HSPC25, HSPC50, HSPC100, HSPC150 and HSPC200 gels determined by FTIR.

Samples	Secondary Structure (%)			
	α-Helix (%)	β-Sheet (%)	β-Turn (%)	Random Coil (%)
HSPC0	33.62 ± 0.47 ^e^	41.84 ± 1.36 ^e^	19.83 ± 1.00 ^a^	4.70 ± 1.19 ^a^
HSPC25	33.27 ± 0.25 ^e^	40.27 ± 1.82 ^de^	20.88 ± 0.90 ^a^	5.58 ± 0.29 ^a^
HSPC50	31.60 ± 1.17 ^d^	39.15 ± 1.37 ^cd^	22.34 ± 1.72 ^ab^	6.57 ± 0.84 ^a^
HSPC100	29.50 ± 0.94 ^c^	37.12 ± 1.43 ^bc^	26.67 ± 2.48 ^bc^	9.04 ± 0.57 ^b^
HSPC150	22.02 ± 0.32 ^b^	35.46 ± 0.15 ^ab^	29.43 ± 0.93 ^c^	12.43 ± 0.93 ^c^
HSPC200	16.67 ± 0.65 ^a^	31.41 ± 0.57 ^a^	34.82 ± 4.27 ^d^	17.09 ± 1.17 ^d^

Note: values with different letters in the same column differ significantly (*p* < 0.05).

## Data Availability

The data presented in this study are available in this article.
